# Supplementary material for: Polycage membranes for precise molecular separation and catalysis
Source: Nat Commun. 2023 May 30;14:3112. doi: 10.1038/s41467-023-38728-7 (PMC10229579; doi:10.1038/s41467-023-38728-7)
Supplement: Supplementary file 1 — Supplementary information [file 41467_2023_38728_MOESM1_ESM.pdf]

Supplementary Information for

Polycage membranes for precise molecular separation and catalysis

Xiang Li[1, 2], Weibin Lin[2,3], Vivekanand Sharma[2,3], Radoslaw Gorecki[1, 2], Munmun Ghosh[2,3], Basem A. Moosa[2,3], Sandra Aristizabal[1, 2], Shanshan Hong[1, 2], Niveen M. Khashab[2, 3]* and Suzana P. Nunes[1, 2, 3, 4]*

[1]Environmental Science and Engineering Program, Biological and Environmental Science and Engineering Division (BESE), [2]Advanced Membranes and Porous Materials (AMPM) Center, [3]Chemistry Program, Chemical Engineering, Physical Science and Engineering Division (PSE), [4]King Abdullah University of Science and Technology (KAUST), 23955-6900 Thuwal, Saudi Arabia

* Corresponding authors : suzana.nunes@kaust.edu.sa; niveen.khashab@kaust.edu.sa

Materials

Trimesoyl chloride (TMC, 98%) and (R,R)-1,2-diaminocyclohexane (98%) and diphenyl-4,4'-dicarbaldehyde (>98%) were procured from TCl chemistry. 1,3,5-triformylbenzene (99%) was obtained from Yanshen Technology Co. Ltd. N1,N1-bis(2-aminoethyl)ethane-1,2-diamine (tren, 96%), palladium (II) acetate (reagent grade, 98%), trifluoroacetic acid (HPLC grade, >99.0%) and sodium borohydride (NaBH4, 99%) were purchased from Sigma-Aldrich. All organic solvents were HPLC grade from VWR Chemicals. All chemicals were used as received without further purification. The porous AAO support (Anodisc TM 25, pore size 0.02 μm) was purchased from GE Healthcare Life Sciences. The porous PAN membrane was obtained from GMT GmbH. Deionized (DI) water (>18 MΩ cm) used in all experiments was filtered through a Millipore Milli-Q water purification system.

Molecular simulation

Molecular modelling of tren and RCC3 cages was constructed by referring to their relevant single crystal structures with CCDC deposition numbers of 1020550 and 1955764, respectively. Protons of amine groups were omitted due to amide reaction during membrane formation. Restrained electrostatic potentials (RESPs) were calculated and assigned using the Multiwfn software after geometry optimization with the ORCA package by density functional theory (DFT) calculations at B3LYP-D3(BJ)/def2-SVP level[1, 2].

Models of monomer molecules are constructed and described with polymer-consistent force field (PCFF). In initial, a ratio of 3 TMC molecules and 1 tren cage, or 4 TMC and 1 RCC cage, is packed in a cubic cell with a dimension of 35 Angstrom at low density of 1.181 g/cm^3 or 1.114 g/cm^3, respectively. The chlorines in TMC molecules and the amino hydrogens in cage molecules are removed. Then carbons of carbonyls in TMC molecules and nitrogen atoms in cage molecules are marked with a tag, followed by polymerization steps between tagged carbons and nitrogen atoms within a cutoff of 6 Angstrom, with energy minimization and MD steps to adjust

molecules. Polymatic is used in generating amorphous polymer models and polymerization[3]. The polymerization cycles were repeated until no pair meeting the bonding criteria was identified. The polymerized structure is finally annealed with a 21-step molecular dynamics equilibration. Unreacted ends of carbonyls and amine are restored to chlorines and hydrogens. LAMMPS is used in both MD step of polymerization and the 21-step relaxation procedure[4]. For analysis, Material Studio is used to analyze accessible surface with a probe of 1 Å radius. Zeo^{++} is used to analyze voids, including void space and pore size distribution[5].

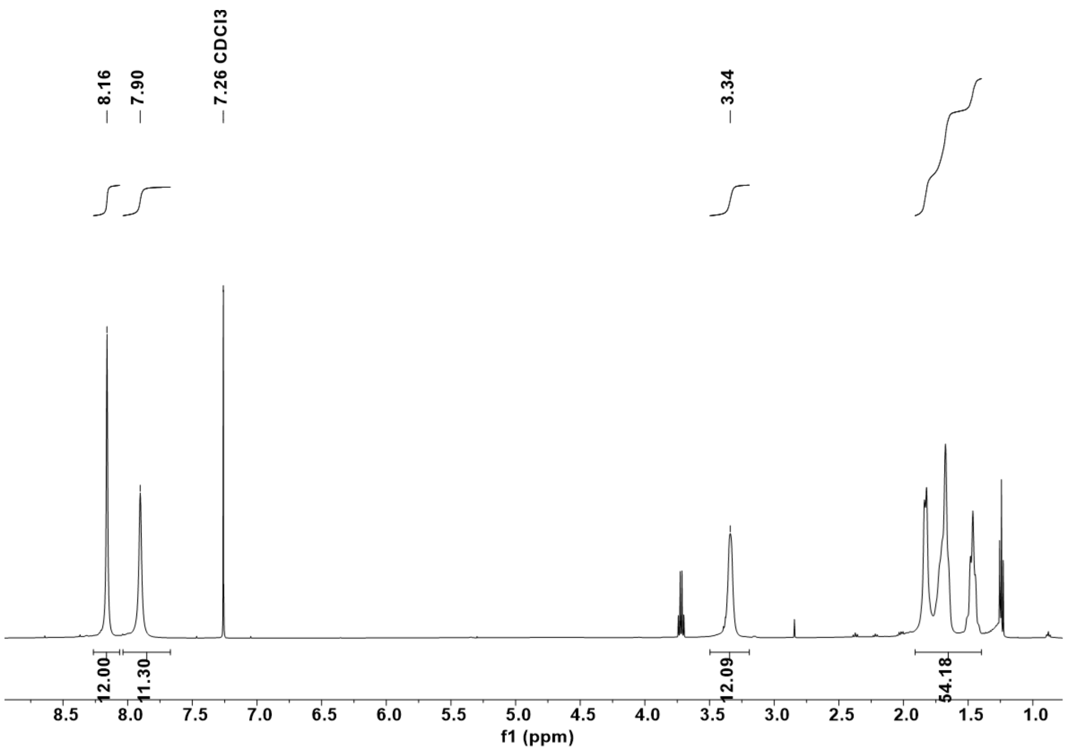

Supplementary Figure 1. ^{1}H NMR of CC3-R ($CDCl_3$, 400 MHz).

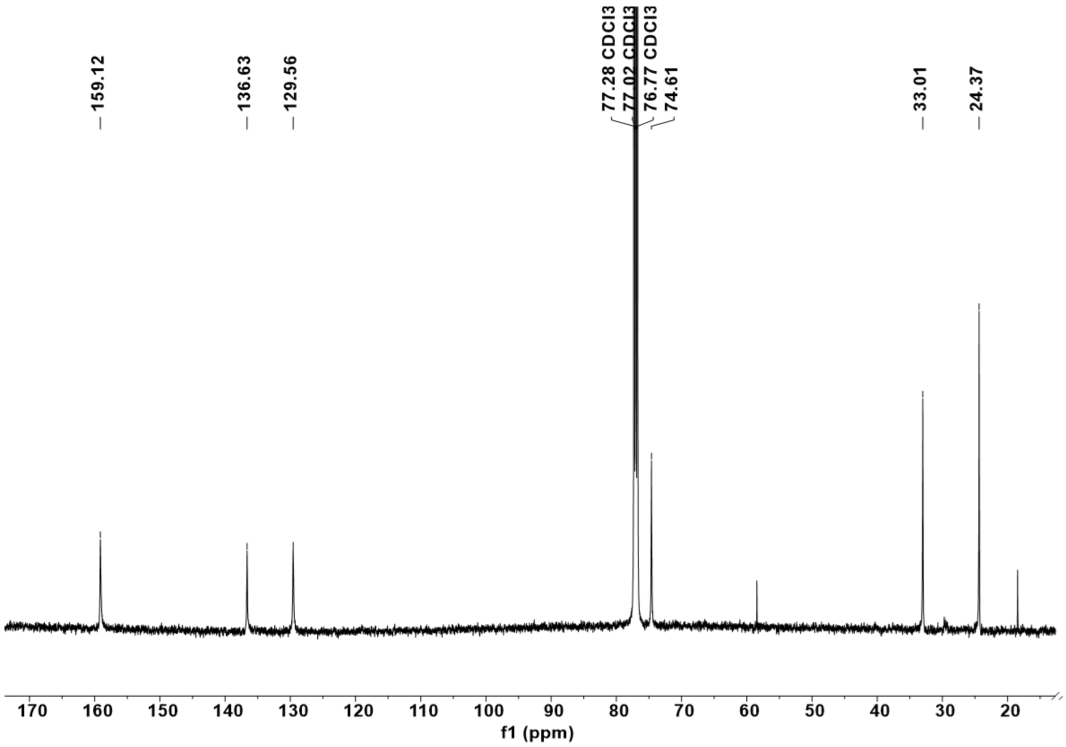

Supplementary Figure 2. ^{13}C NMR of CC3-R ($CDCl_3$, 125 MHz).

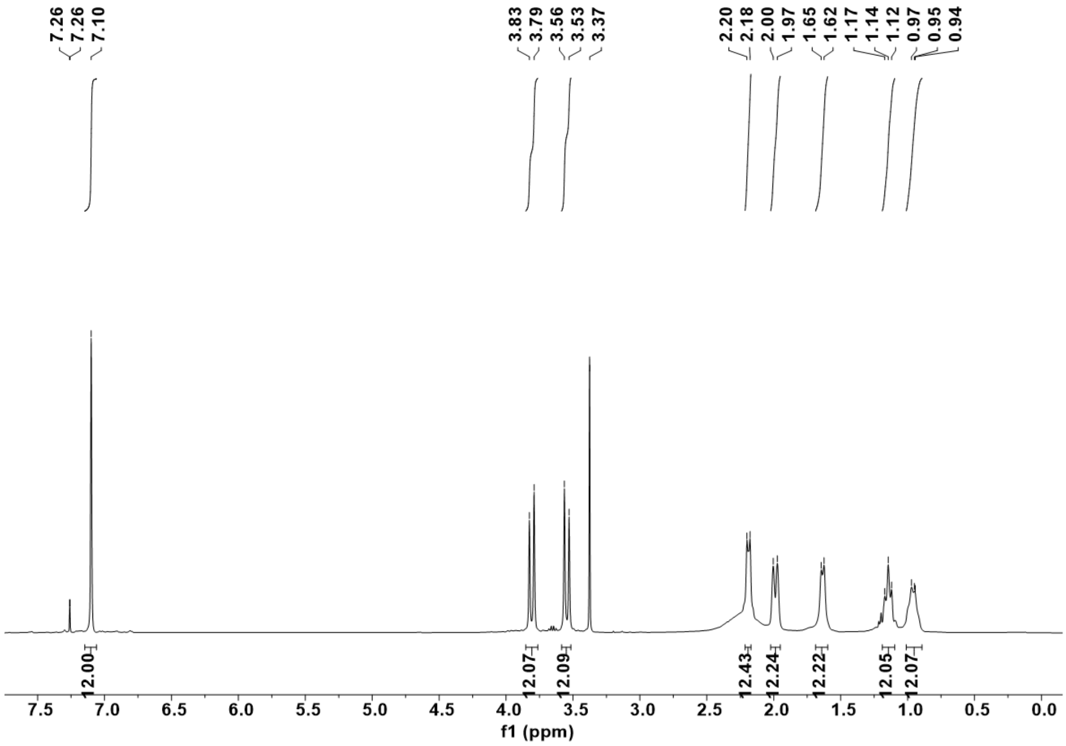

Supplementary Figure 3. ^{1}H NMR of purified RCC3 ($CDCl_3$, 400 MHz).

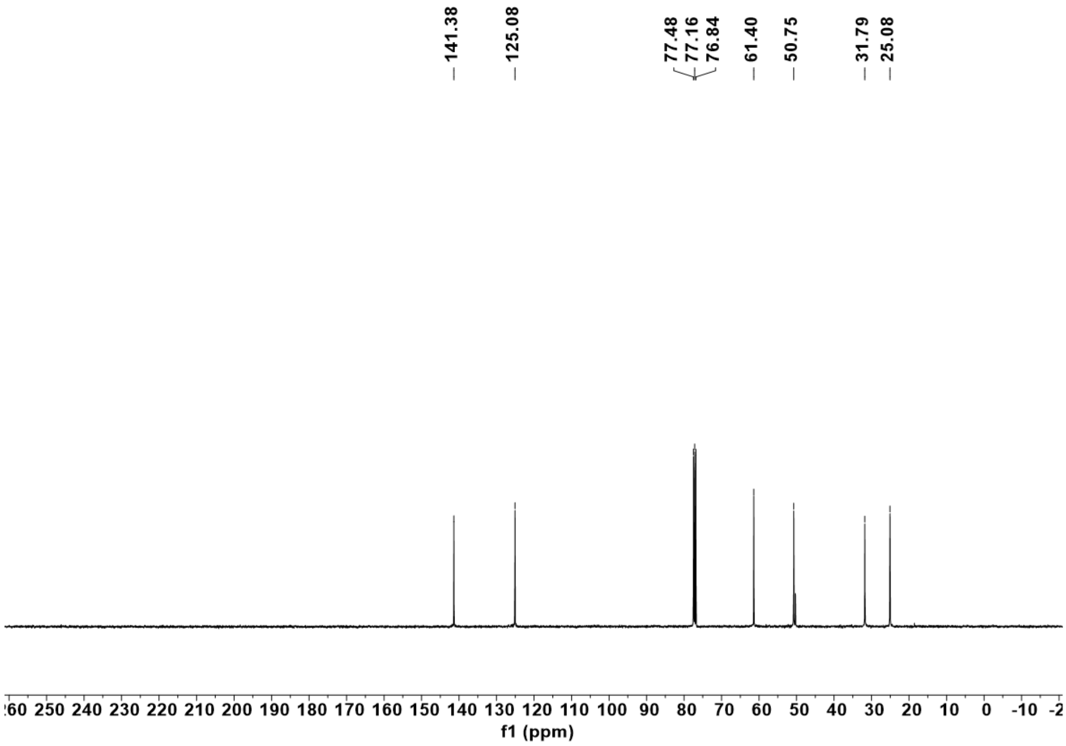

Supplementary Figure 4. ^{13}C NMR of purified RCC3 ($CDCl_3$, 125 MHz).

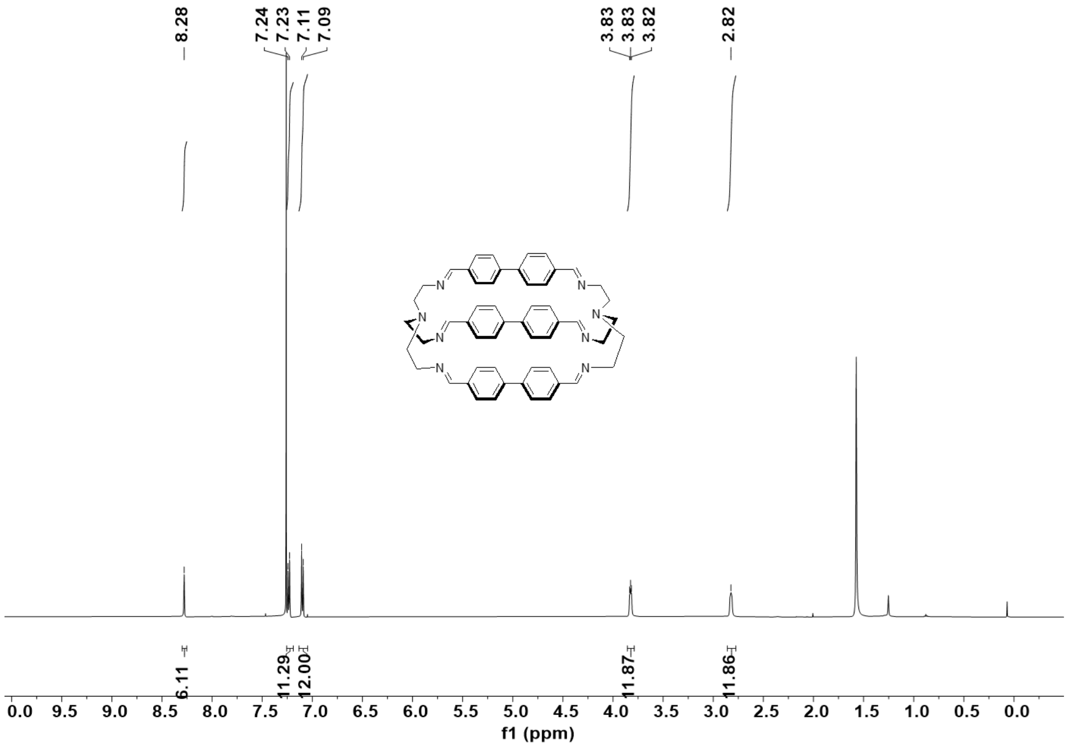

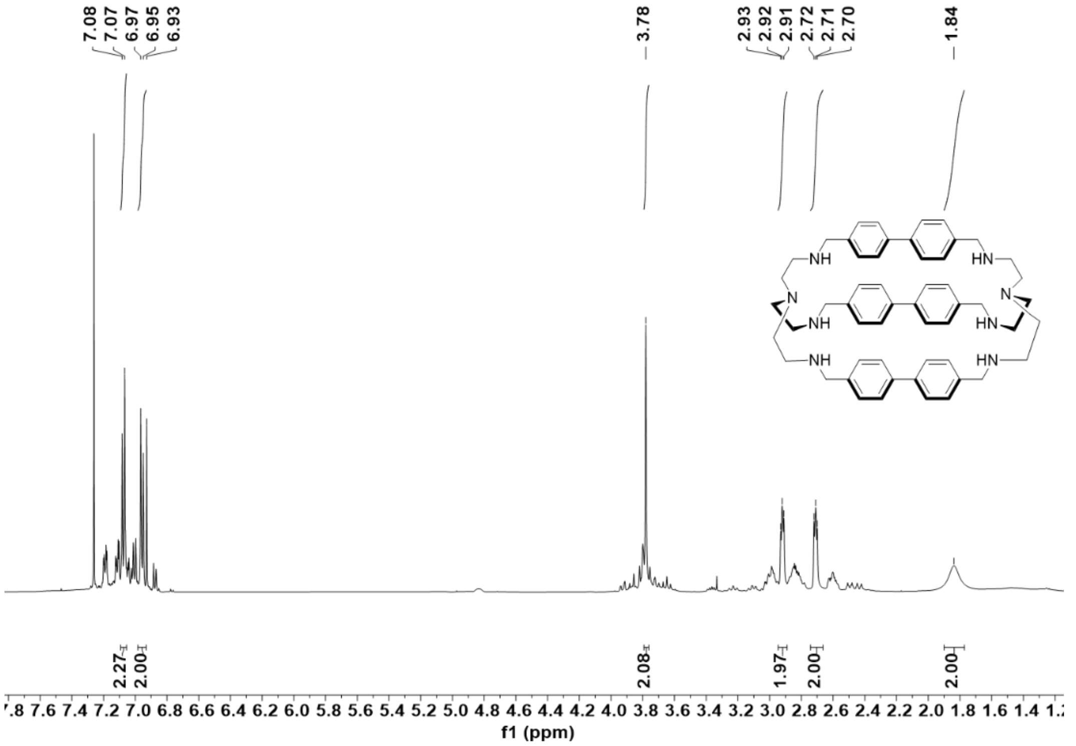

Supplementary Figure 5. ^{1}H NMR of tren-cage imine and amine-functionalized tren-cage ($CDCl_3$, 400 MHz).

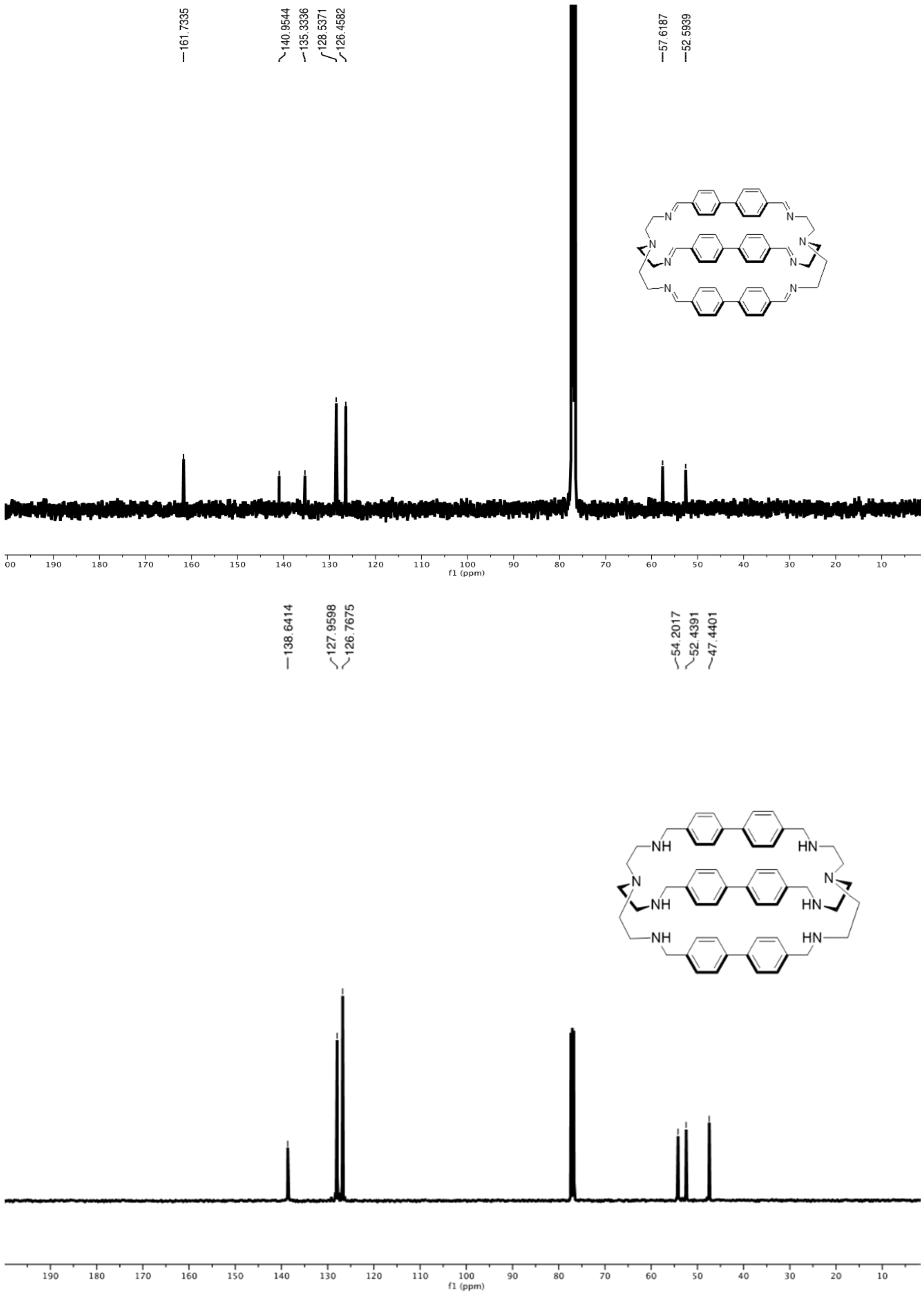

Supplementary Figure 6. ^{13}CNMR of tren-cage imine and amine-functionalized tren-cage ($CDCl_3$, 125 MHz).

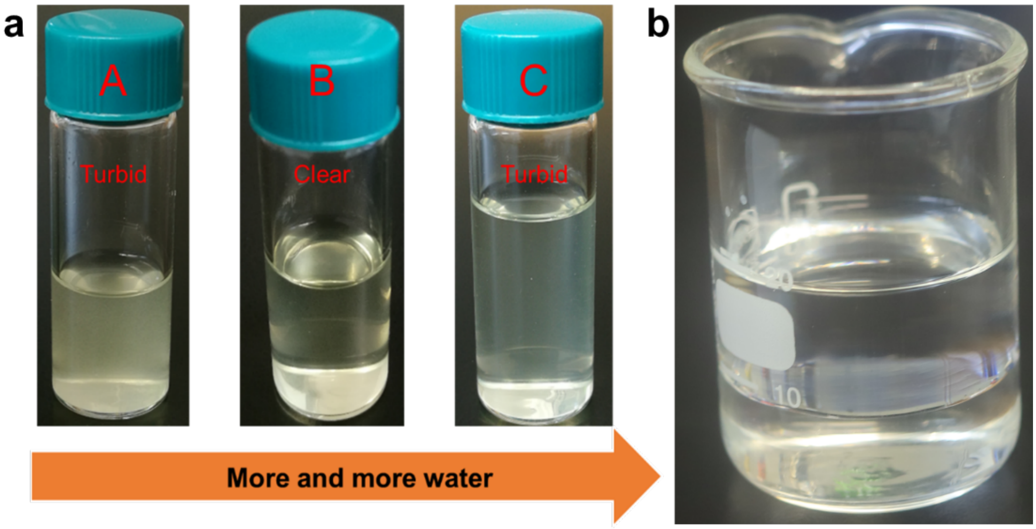

Supplementary Figure 7. **a**, Photographs of tren-cage solution in TFE after adding an increasing quantity of water; **b**, Tren-TFE nanofilm formed at the hexane/TFE-H_2O interface.

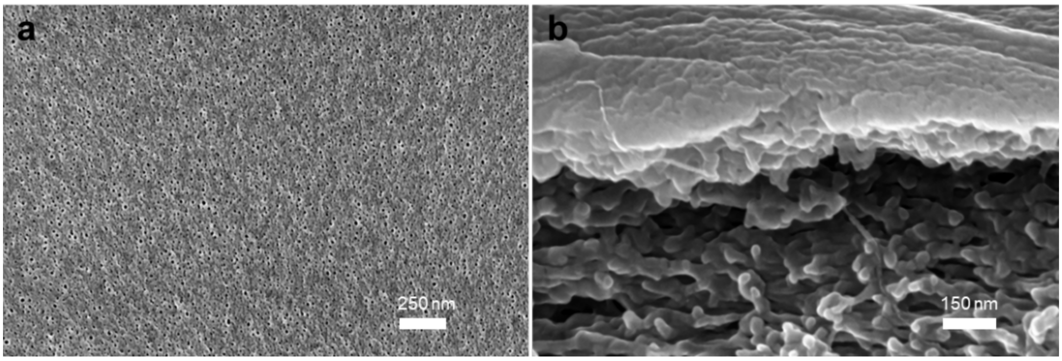

Supplementary Figure 8. SEM images of PAN substrate. **a**, Surface; **b**, Cross-section.

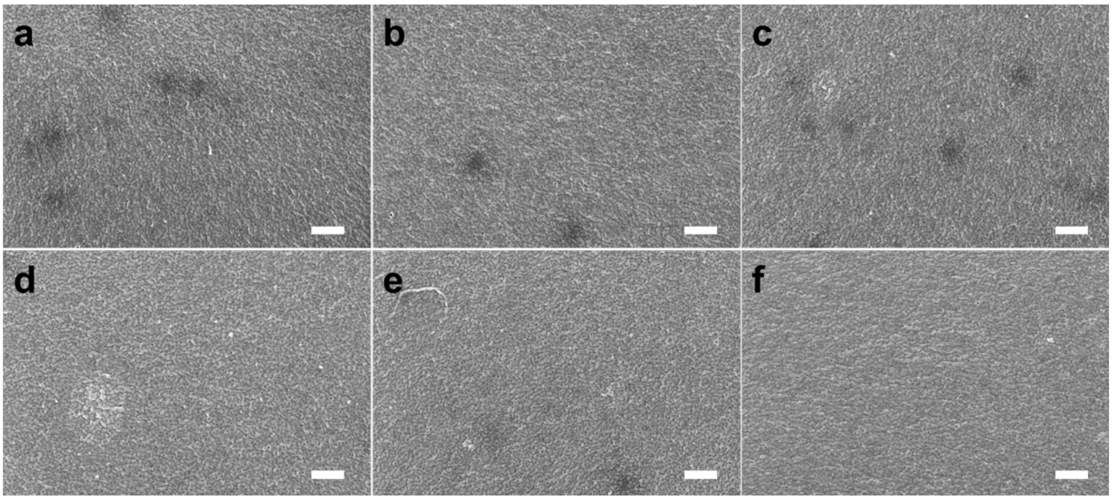

Supplementary Figure 9. SEM images of the surface of tren-acid membranes with 0.05 wt% tren-cage in the aqueous phase and 0.1 wt/v% TMC in hexane phase. Reaction times: **a**, 10 s; **b**, 30 s; **c**, 1 min; **d**, 2 min; **e**, 5 min; **f**, 10 min. Scale bar: 250 nm. TFC membranes prepared by IP on PAN support.

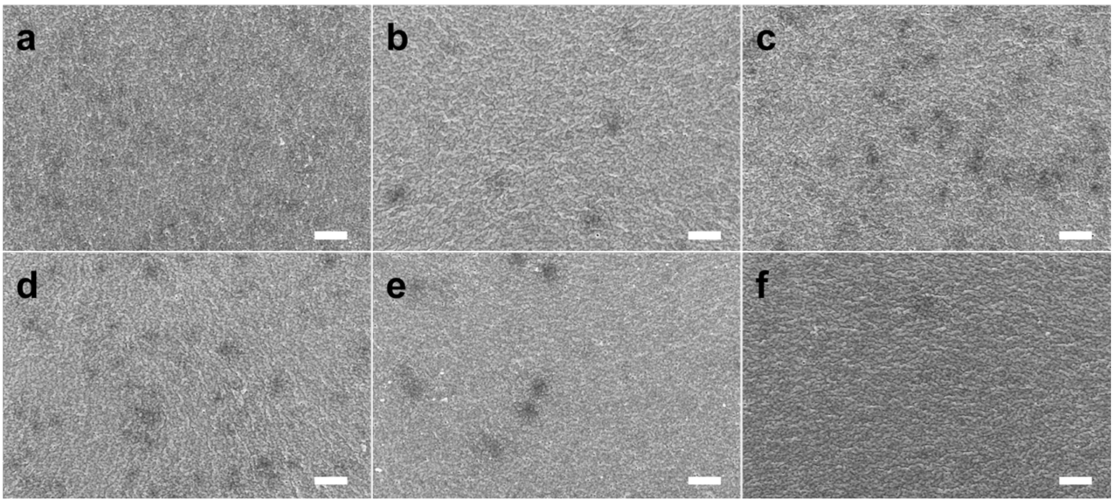

Supplementary Figure 10. SEM images of the surface of RCC3-acid membranes with 1 wt% RCC3 in the aqueous phase and 0.1 wt/v% TMC in hexane phase. Reaction times: **a**, 10 s; **b**, 30 s; **c**, 1 min; **d**, 2 min; **e**, 5 min; **f**, 10 min. Scale bar: 250 nm. TFC membranes prepared by IP on PAN support.

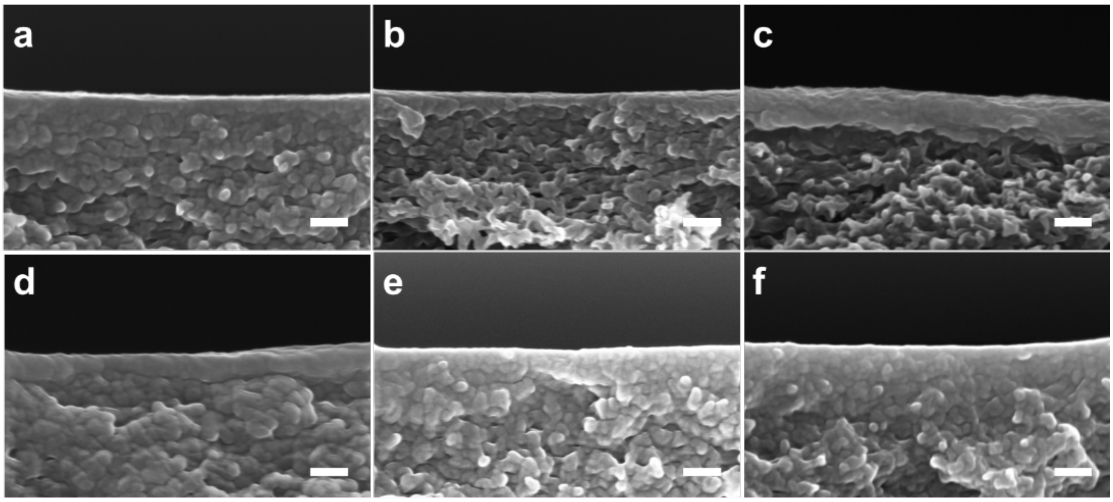

Supplementary Figure 11. SEM images of the cross-section of tren-acid membranes with 0.05 wt% tren-cage in the aqueous phase and 0.1 wt/v% TMC in hexane phase. Reaction times: **a**, 10 s; **b**, 30 s; **c**, 1 min; **d**, 2 min; **e**, 5 min; **f**, 10 min. Scale bar: 150 nm. TFC membranes prepared by IP on PAN support.

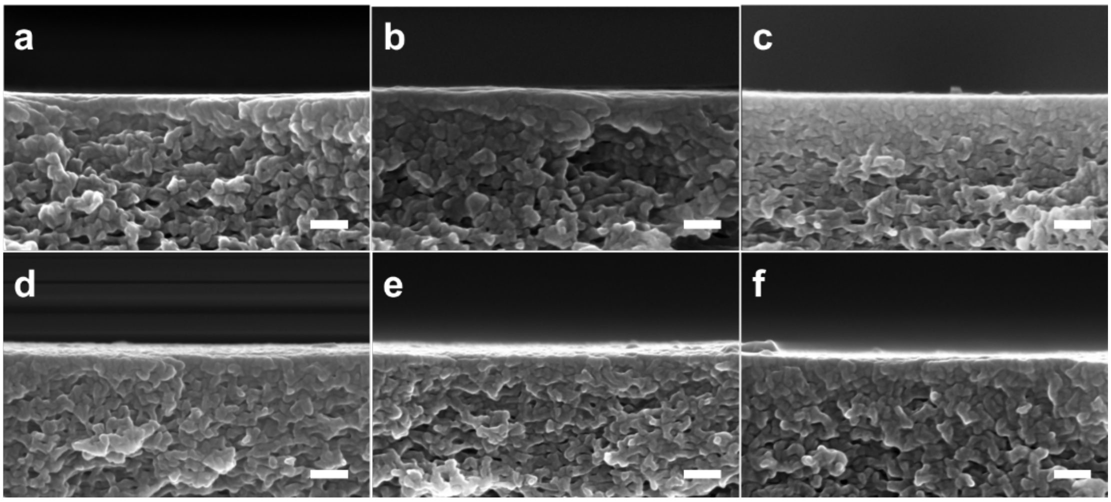

Supplementary Figure 12. SEM images of the cross-section of RCC3-acid membranes with 1 wt% RCC3 in the aqueous phase and 0.1 wt/v% TMC in hexane phase. Reaction times: **a**, 10 s; **b**, 30 s; **c**, 1 min; **d**, 2 min; **e**, 5 min; **f**, 10 min. Scale bar: 150 nm. TFC membranes prepared by IP on PAN support.

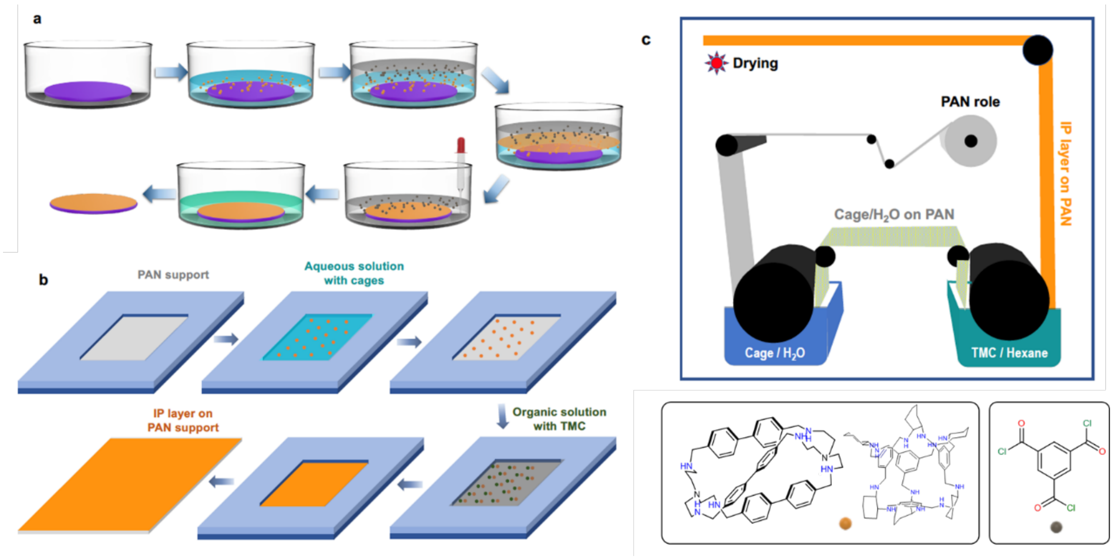

Supplementary Figure 13. Schematic of polycage IP nanofilms fabrication. a, Free-standing between organic and aqueous phase. Silica wafer at the bottom of petri dish, layer formation at the interface, liquid collection via a syringe to lay the nanofilm onto the substrate, washing with hexane. **b**, TFC membrane preparation on PAN support at lab scale using frames. **c**, TFC membrane preparation on PAN support role in a continuous machine.

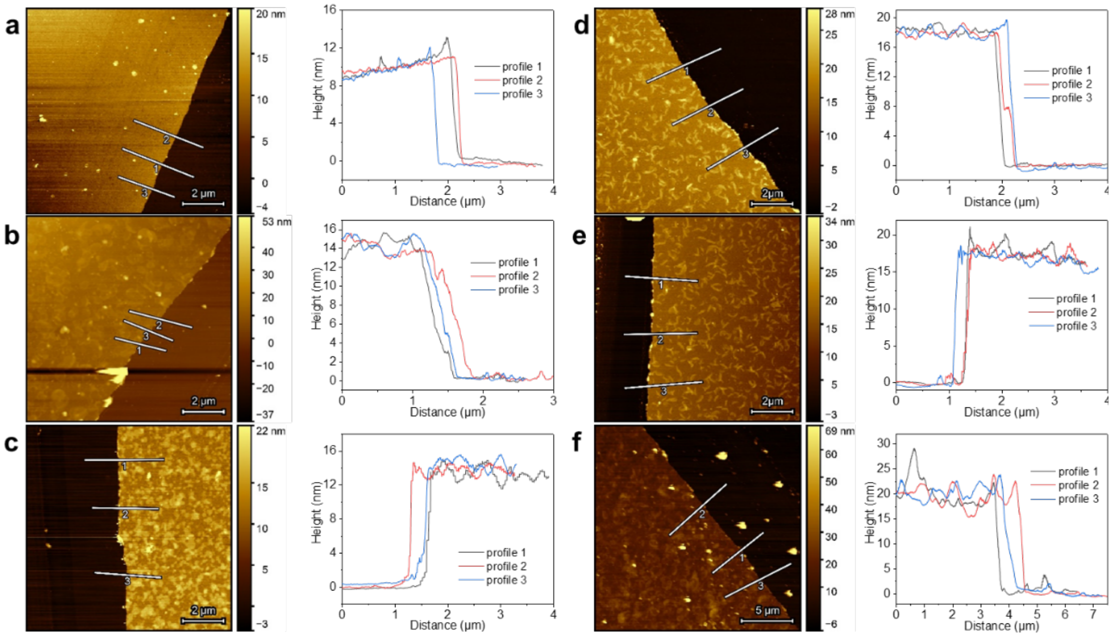

Supplementary Figure 14. AFM images and height profile of freestanding polycage-acid nanofilms, prepared with 0.05 % tren-cage with different reaction times: **a**, 10 s, **b**, 30 s, **c**, 1 min, **d**, 2 min, **e**, 5 min, and **f**, 10 min. Free-standing nanofilms on silicon.

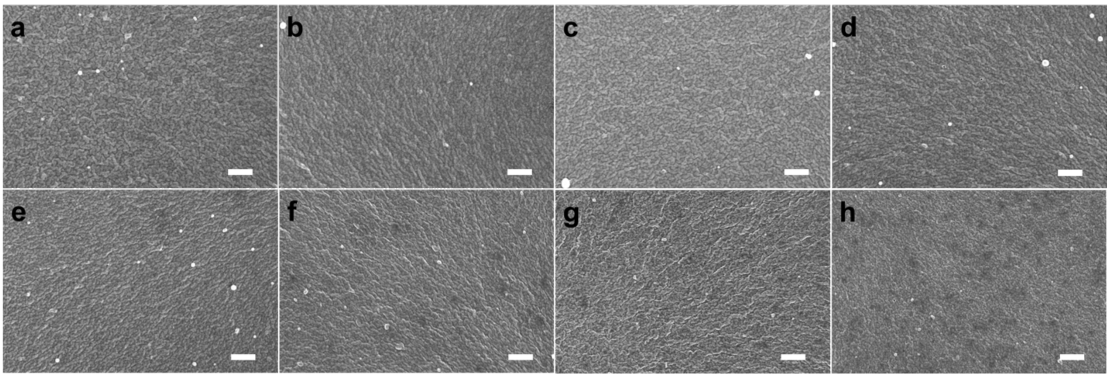

Supplementary Figure 15. SEM images of the surface of tren-tfe membranes with 0.3 wt% tren-cage in the TFE-H_2O phase and 10s of reaction time. TMC concentration in hexane (wt/v%): **a**, 0.001; **b**, 0.005; **c**, 0.025; **d**, 0.05; **e**, 0.07; **f**, 0.1; **g**, 0.15; **h**, 0.2. Scale bar: 250 nm. TFC membranes prepared by IP on PAN support.

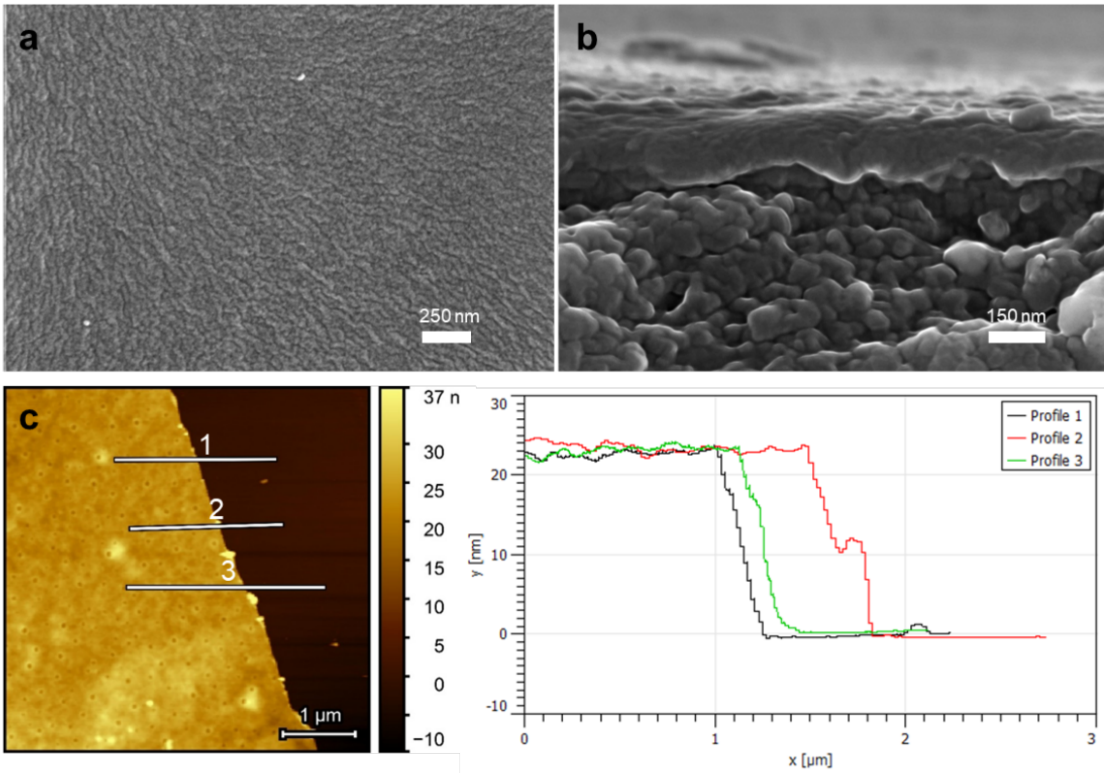

Supplementary Figure 16. RCC3-TFE-0.005 membrane morphology. SEM images of the **a**, SEM surface image; **b**, SEM cross-section image. **c**, AFM image and height profiles of self-standing layer. **a, b** TFC membranes prepared by IP on PAN support.

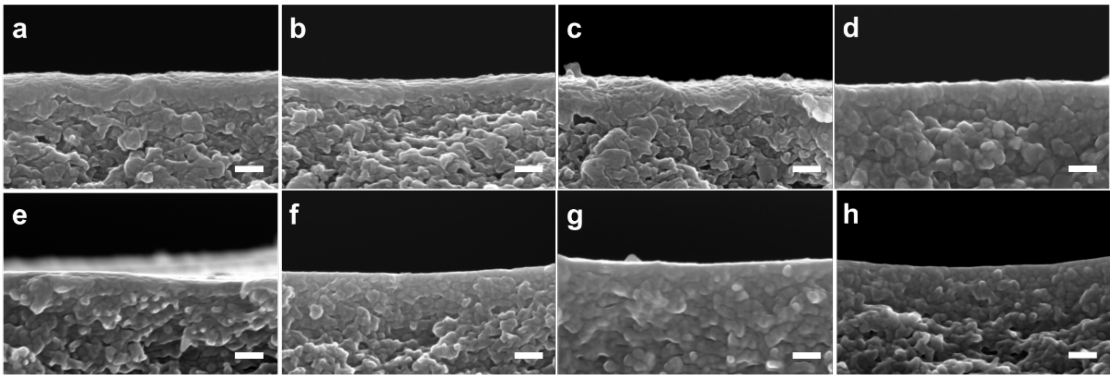

Supplementary Figure 17. SEM images of the cross-section of tren-TFE membranes with 0.3 wt% tren-cage in the TFE-H_2O phase and 10s of reaction time. TMC concentration in hexane (wt/v%): **a**, 0.001; **b**, 0.005; **c**, 0.025; **d**, 0.05; **e**, 0.07; **f**, 0.1; **g**, 0.15; **h**, 0.2. Scale bar: 150 nm. TFC membranes prepared by IP on PAN support.

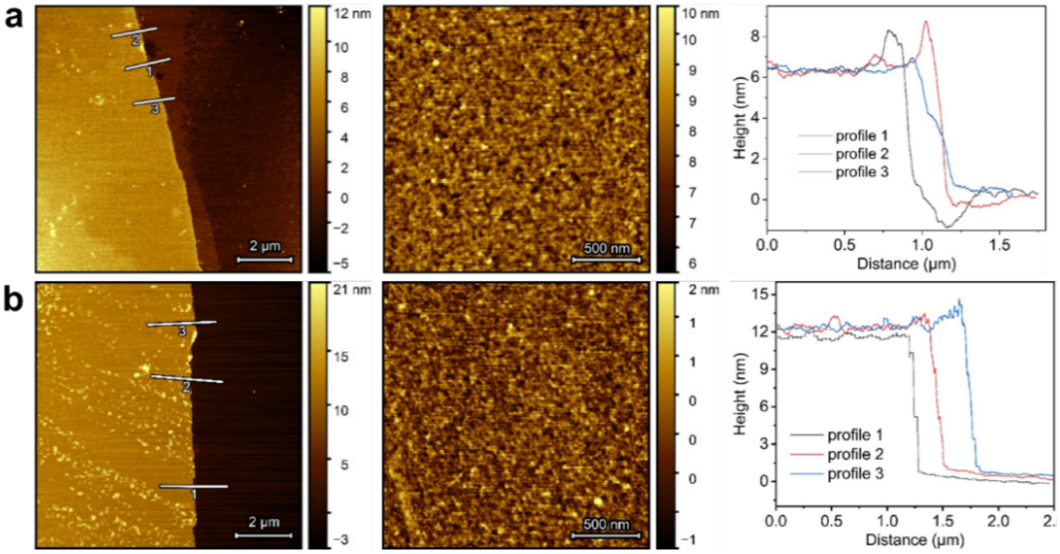

Supplementary Figure 18. AFM images and height profiles of free-standing RCC3-acid nanofilms on silicon, prepared with 1wt% RCC3 with different reaction times: **a**, 10 s; **b**, 10 min. TMC concentration in hexane is 0.1 wt/v%.

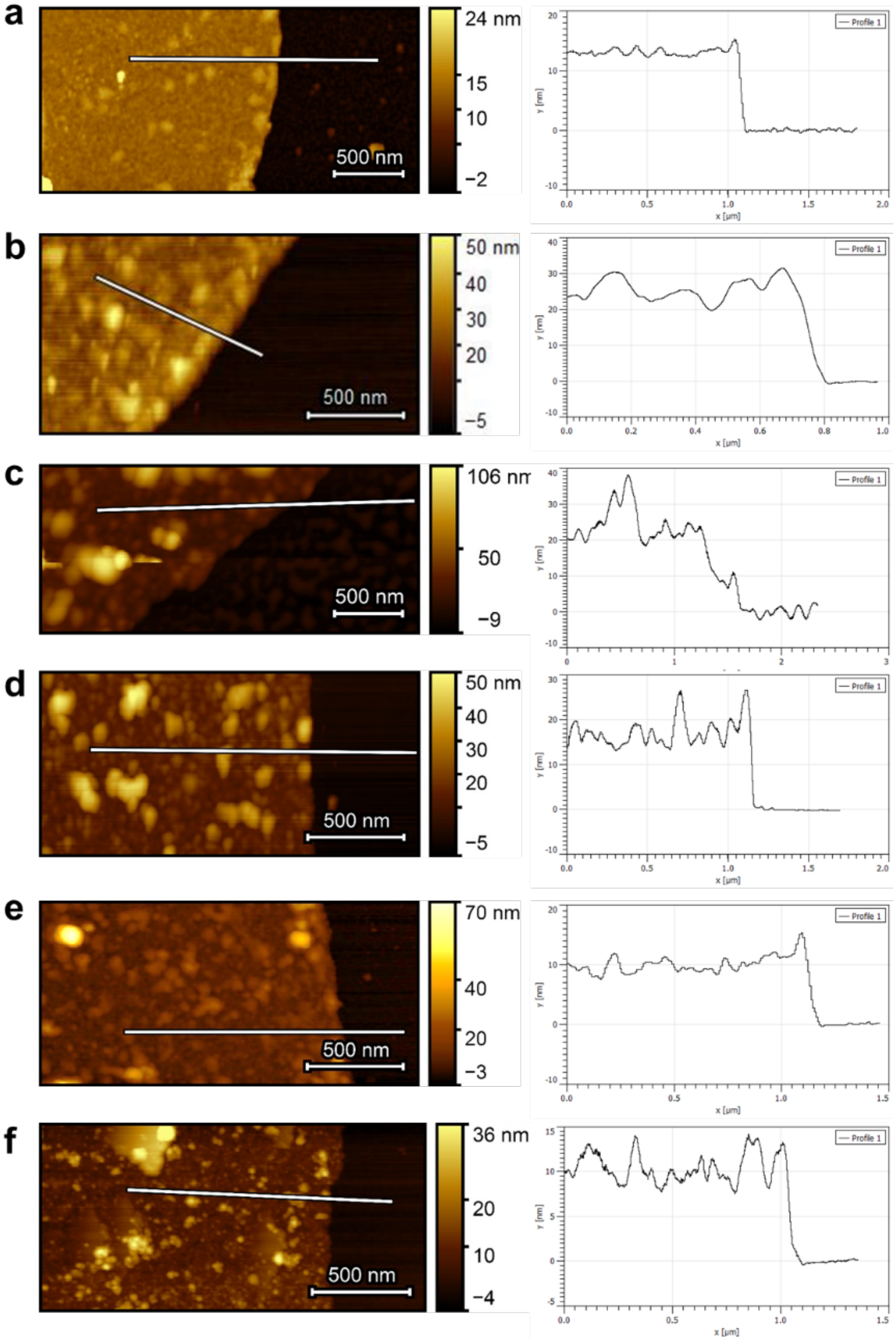

Supplementary Figure 19. 2D AFM images and height profile of free-standing polycage nanofilms on silicon, prepared with 0.3 wt/v% tren-cage in TFE-H_2O phase with different TMC concentration in hexane: **a**, 0.005 **b**, 0.025, **c**, 0.05, **d**, 0.07, **e**, 0.1, and **f**, 0.15. Reaction time is 10 s.

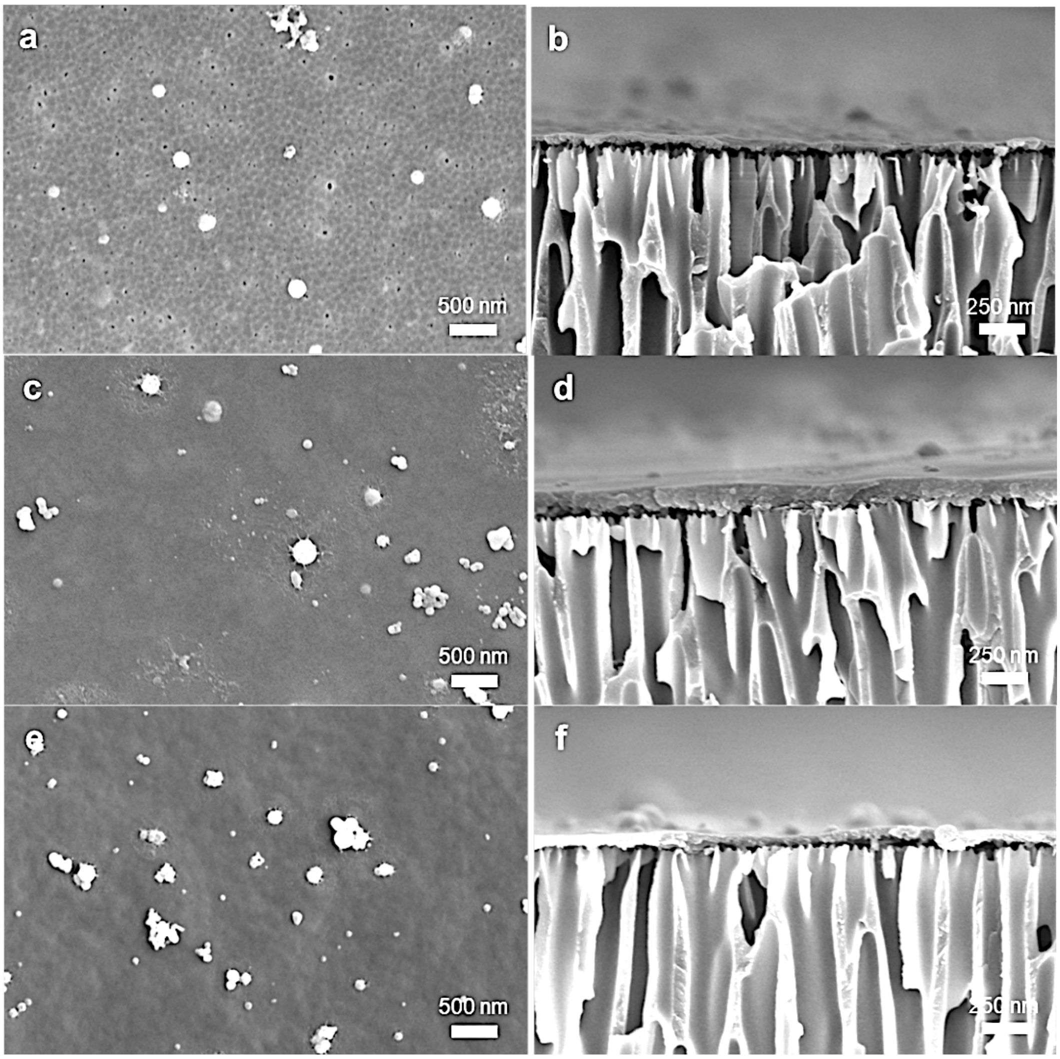

Supplementary Figure 20. SEM images of tren-based free-standing nanofilms prepared with 0.5 wt/v% tren-cage in the TFE-H_2O phase collected on alumina porous support. a, c, e, Surface; **b, d, f,** Cross-section. TMC concentration in hexane (wt/v%): **a, b**, 0.005; **c, d,** 0.015; **e**, **f,** 0.025.

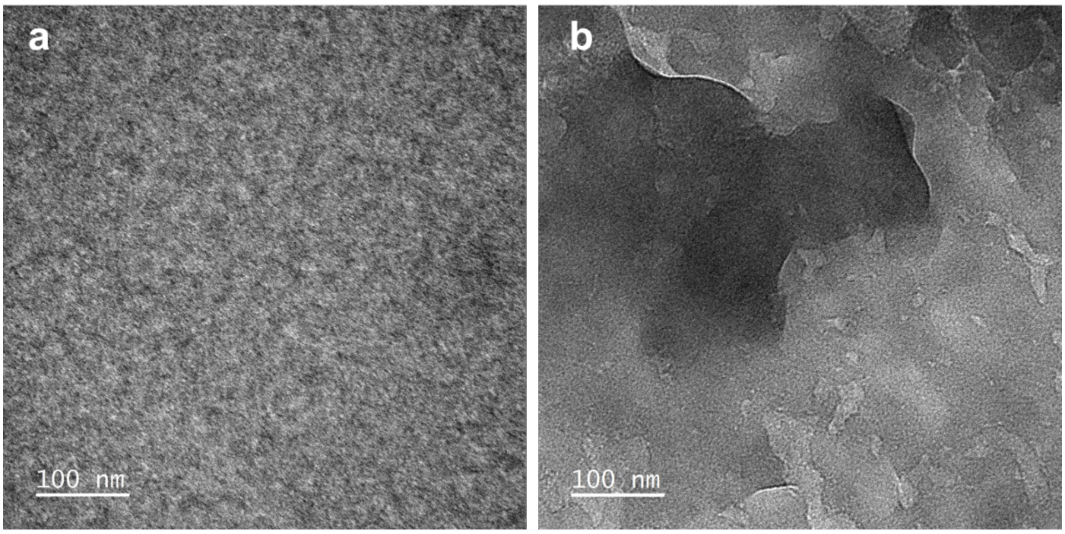

Supplementary Figure 21. TEM micrographs. a, tren-TFE nanofilm; **b,** RCC3-TFE nanofilm. Free-standing films on TEM grids.

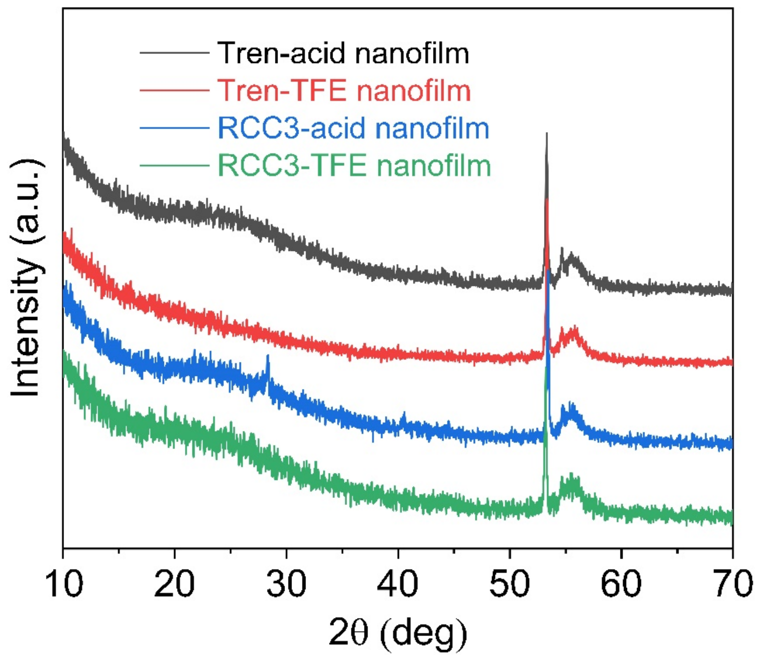

Supplementary Figure 22. XRD of polycage nanofilms. Free-standing films on silicon.

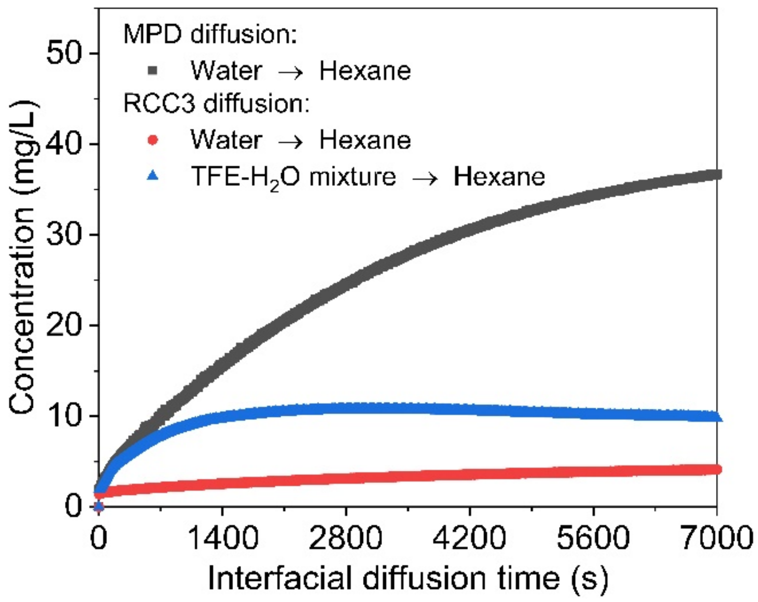

Supplementary Figure 23. Monomer concentration detected from the water-hexane interface and the TFE/H_2O mixture–hexane interface via UV spectroscopy versus interfacial diffusion time. TFC membranes prepared by IP on PAN support.

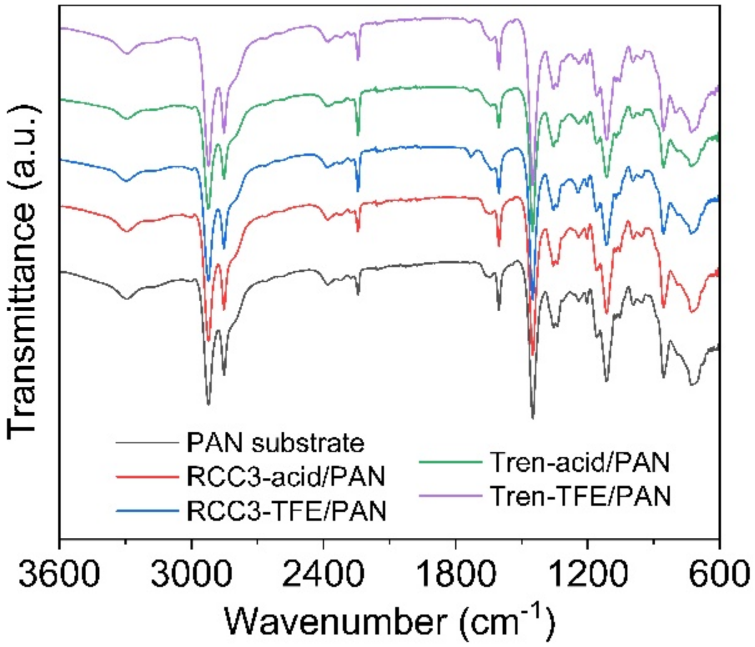

Supplementary Figure 24. ATR-FTIR spectra of PAN support and polycage TFC membranes.

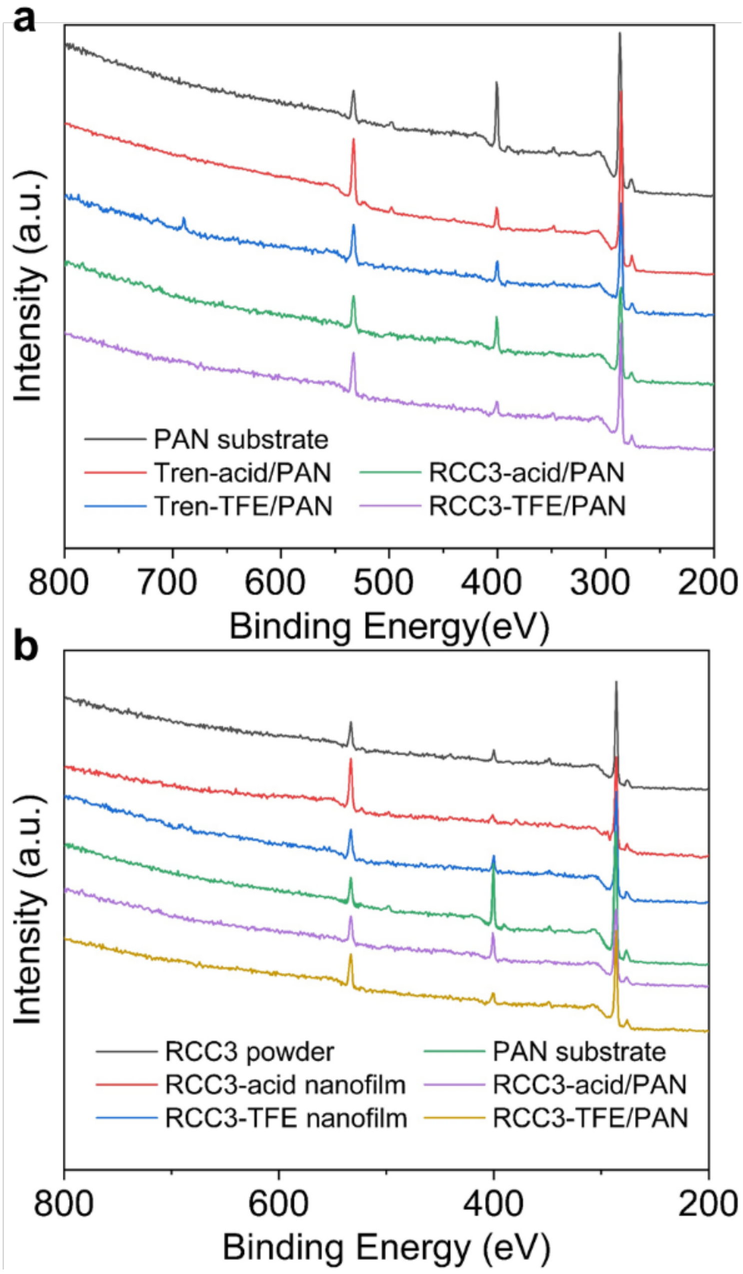

Supplementary Figure 25. XPS survey spectra of PAN substrate, RCC3 power, polycage TFC composite membranes. a, Tren (TFC membranes prepared by IP on PAN support); **b,** RCC3 (free-standing nanofilms on gold-coated steel substrates).

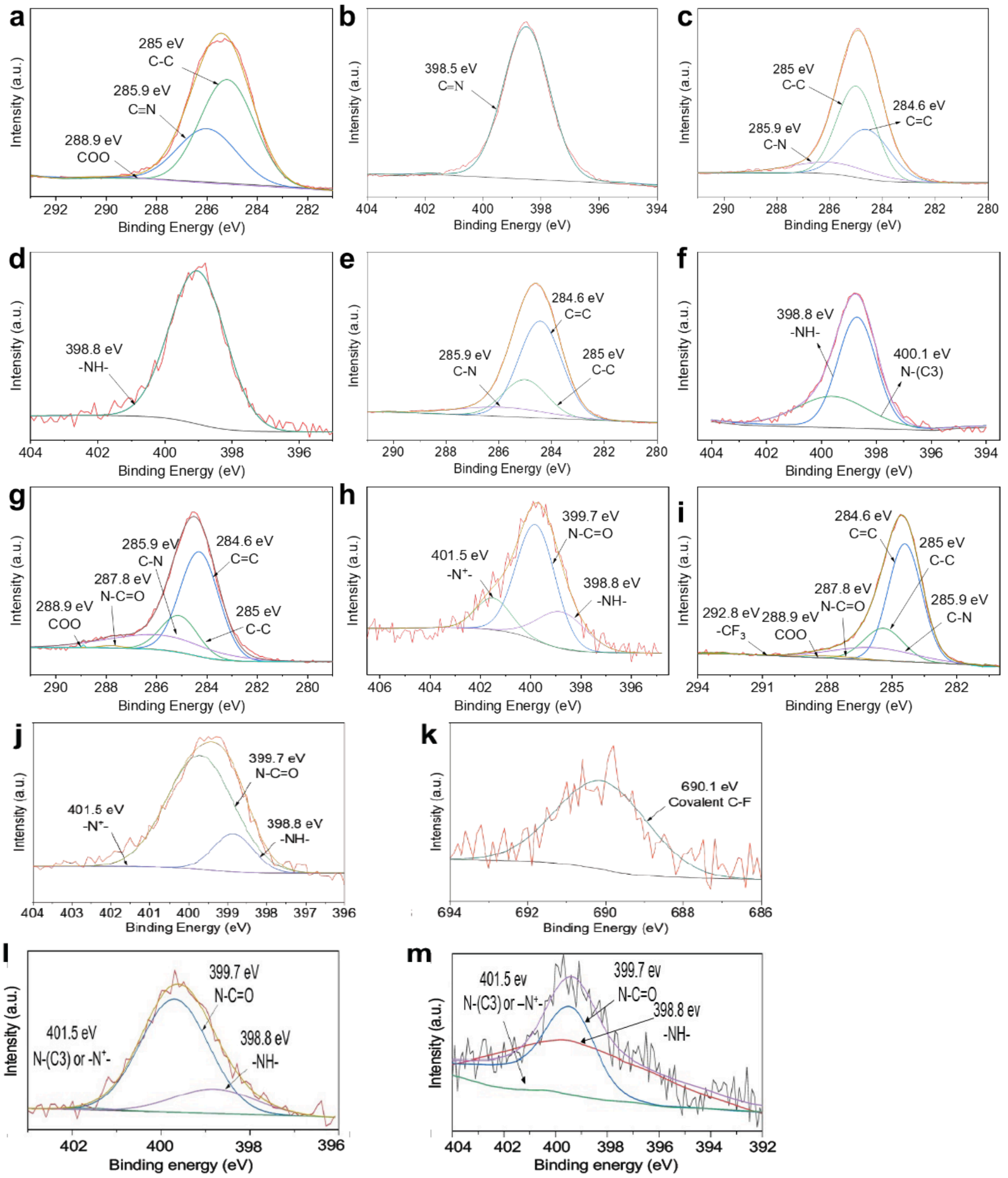

Supplementary Figure 26. XPS survey spectra. a, b, PAN support; **c, d,** RCC3 powder; **e, f,** tren-cage powder; **g, h,** RCC3-acid nanofilm; **i, j, k,** RCC3-TFE nanofilm. **a, c, e, g, I,** C 1s spectra; **b, d, f, h, j,** N1s spectra; **k,** F1s spectra; **l**, **m**, N1s spectra of tren-acid with reaction time 30 min and 10 s. **e-m** Free-standing nanofilms on gold-coated steel substrates.

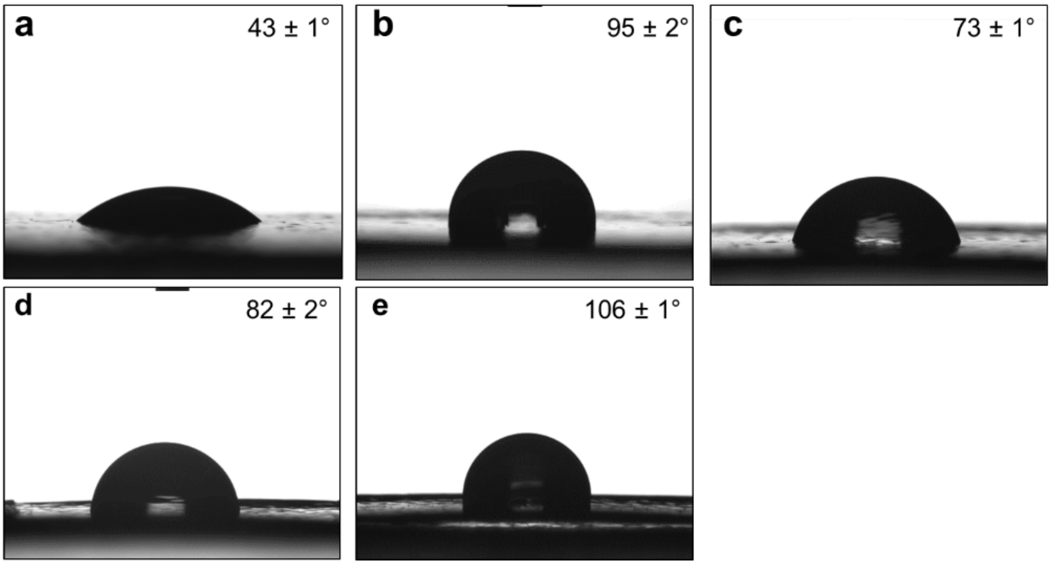

Supplementary Figure 27. Water contact angles. **a**, PAN support; **b**, tren-acid membrane; **c**, tren-TFE membrane; **d**, RCC3-acid membrane; **e**, RCC3-TFE membrane. TFC membranes prepared by IP on PAN support.

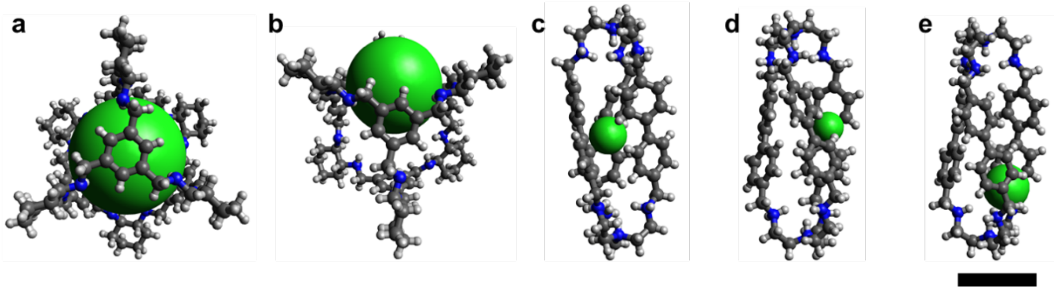

Supplementary Figure 28. Possible transport channels through cages (described by green ball). **a**, Cavity size of 15 Å; **b**, window size of 12.4 Å for RCC3-cage; **c**, cavity size of 4.7Å; **d,e** window size of 3.9 Å and 6.0 Å for tren-cage. Scale bar is 10 Å.

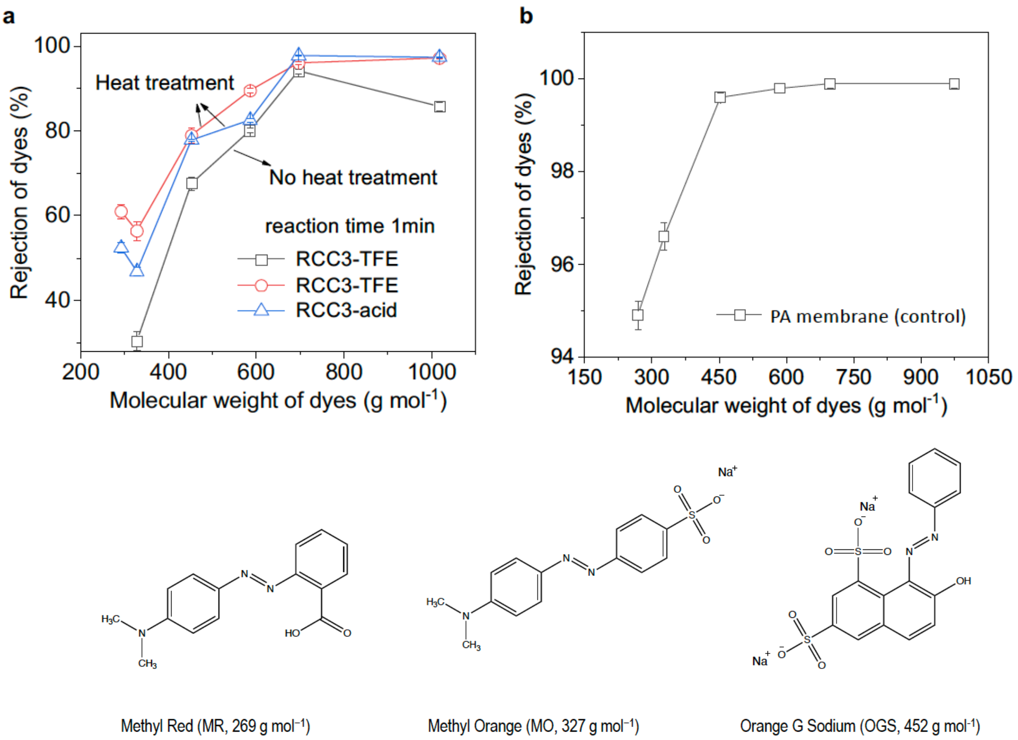

Supplementary Figure 29. Dyes rejection. **a**, RCC3 membranes before/after heat treatment at 80 °C for 10 min. **b**, Control membrane prepared from 2 wt% MPD aqueous solution and 0.1 wt/v% TMC solution in hexane, reaction time of 1 min, followed by 5 min treating at 80 °C. Chemical structures of dyes with molecular weight smaller than 500 g mol^{-1}. TFC membranes prepared by IP on PAN support.

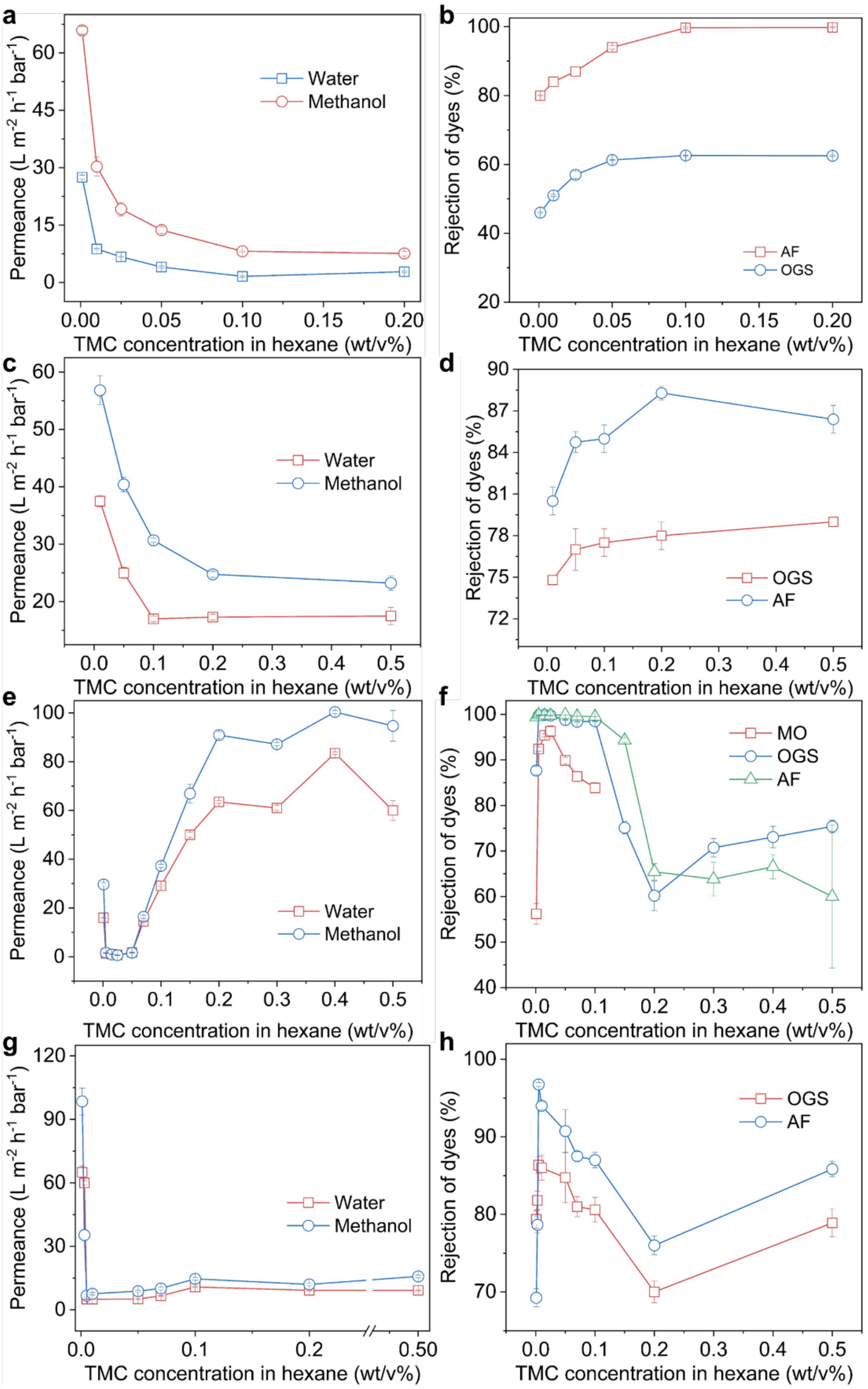

Supplementary Figure 30. Permeance and dye rejections by different membranes prepared with different TMC concentrations in hexane: **a, b**, tren-acid (reaction time 10 s); **c, d**, RCC3-acid (reaction time 1 min); **e, f,** tren-TFE (reaction time 10s); **g, h,** RCC3-TFE (reaction time 10s). TFC membranes prepared by IP on PAN support.

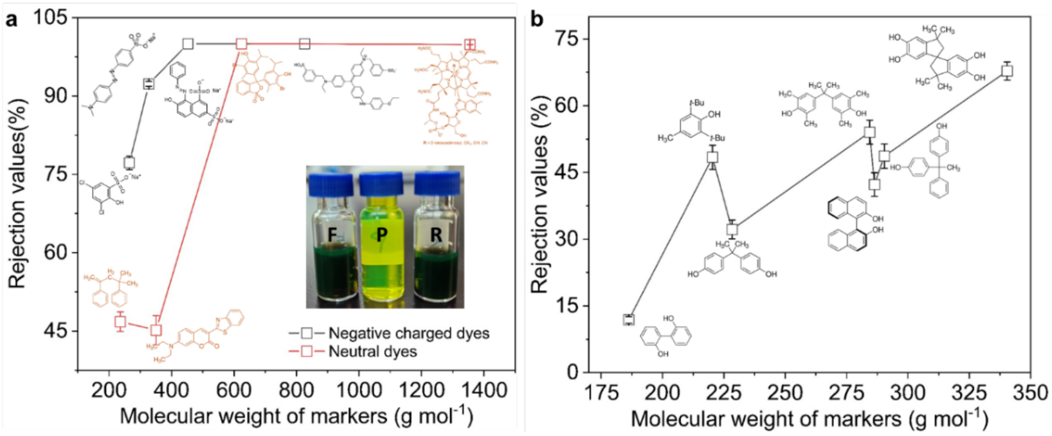

Supplementary Figure 31. Molecular separation performance in organic mixtures with tren-acid-(reaction time 10 s) membranes on PAN support. **a**, Markers with negative (black line) and neutral (red line) charge in methanol, and 10 µM for each composition. Inset, Photos of feed, permeate and retentate solutions within testing. **b**, Phenolic mixture in methanol and 10 µM for each composition. Nanofiltration was conducted in dead-end filtration system at 20 ^{o}C under 5 bar. Rejection versus molecular weight of compounds: Coomassie brilliant blue R 250 (BBR, 826 g mol^{-1}), Orange G Sodium (OGS, 452 g mol^{-1}), Methyl Orange (MO, 327 g mol^{-1}), sodium 3,5-dichloro-2-hydroxybenzenesulfonate (DHBS, 260 g mol^{-1}); Vitamin B12 (VB12, 1355 g mol^{-1}), bromothymol Blue (BrB, 624 g mol^{-1}), Coumarin 6 (C6, 350 g mol^{-1}); 2,2-biphenol (186.2 g mol^{-1}), 2,6-di-tert-butyl-4-methylphenol (220.4 g mol^{-1}), bisphenol A (228.3 g mol^{-1}), 4,4‘ isopropylidenebis (284.4 g mol^{-1}), (R)-(+)-1,1’-Bi-2-naphthol (286.3 g mol^{-1}), 4,4’-(1 phenylethylidene)bisphenol (290.4 g mol^{-1}), 3,3,3’,3’-tetramethyl-1,1’-spirobiindane-5,5’,6,6’-tetraol (340.4 g mol^{-1}). Composition analysis was conducted with HPLC system. TFC membranes prepared by IP on PAN support.

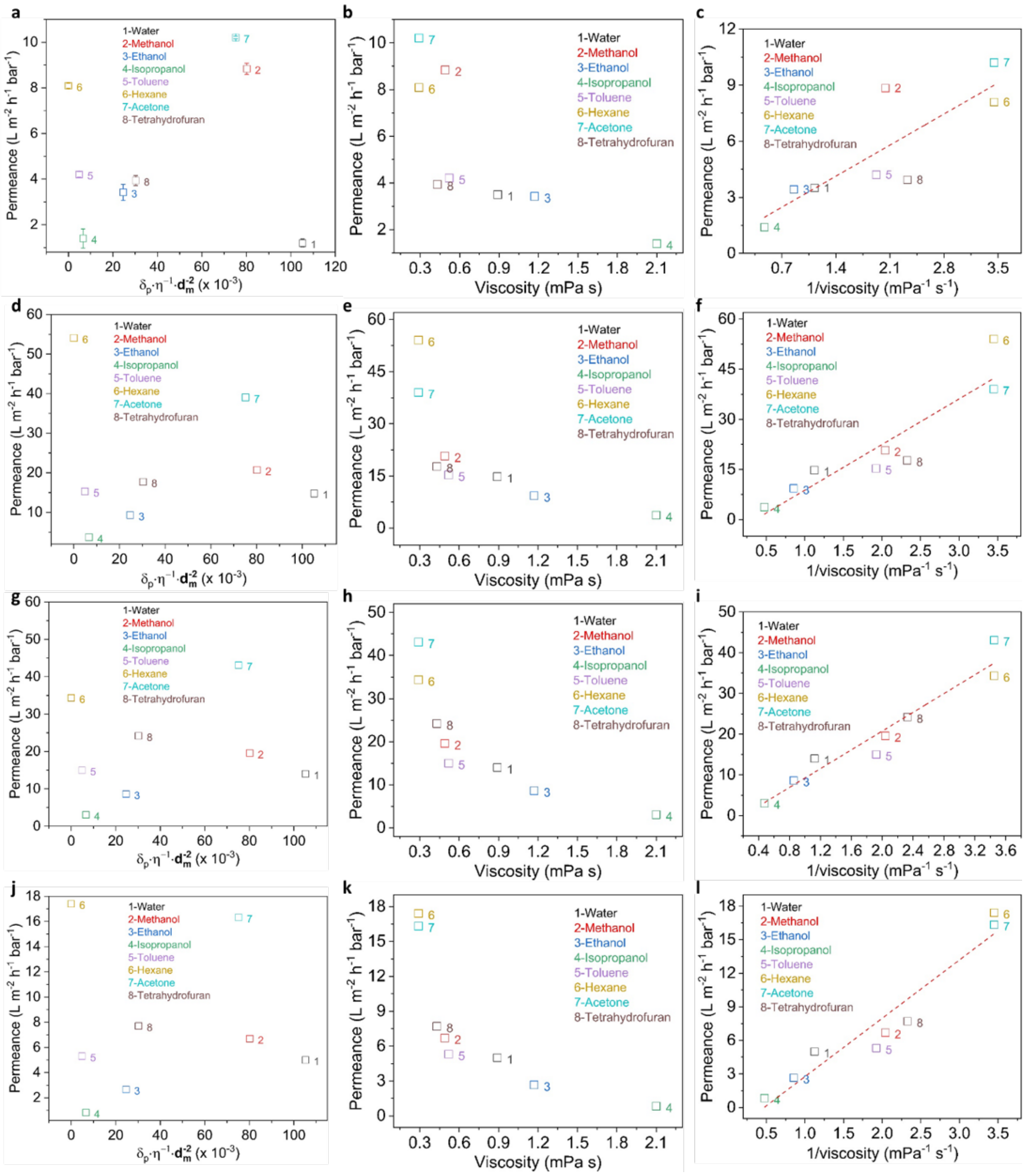

Supplementary Figure 32. Solvents permeance vs viscosity and inverse viscosity through **a, b, c**, tren-acid (reaction time 10 s) membrane, **d, e, f,** tren-TFE-0.07 membrane, **g, h, I,** RCC3-acid (reaction time 5 min) membrane and **j, k, l,** RCC3-TFE-0.005. TFC membranes prepared by IP on PAN support. The calculations were based on the Hansen solubility parameters and the physical properties of the organic solvents[6, 7, 8].

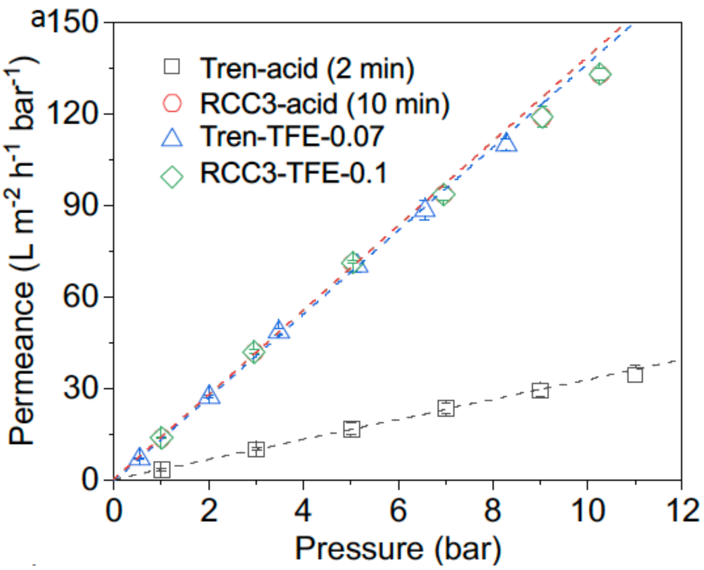

Supplementary Figure 33. Pure methanol permeance vs applied pressure for polycage TFC membranes. TFC membranes prepared by IP on PAN support.

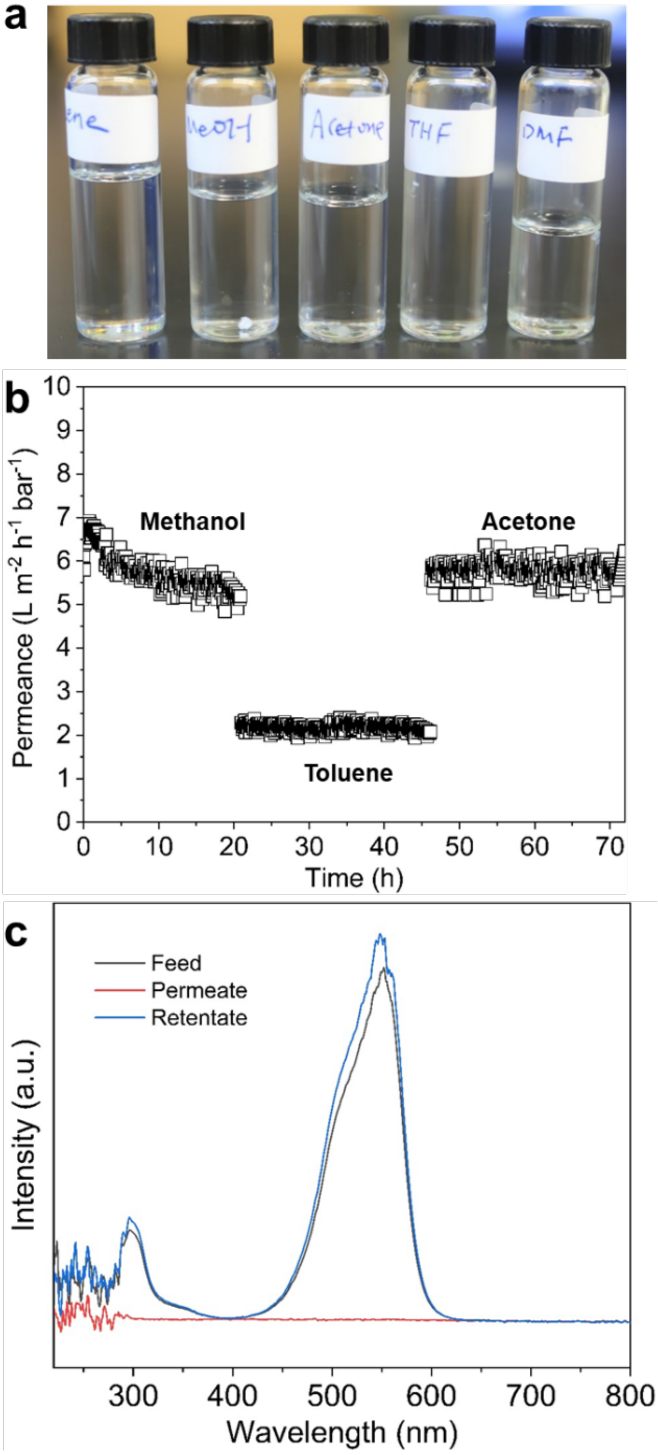

Supplementary Figure 34. a, Tren-acid nanofilms after immersion in various organic solvents for 7 days. **b**, The long-term stability of tren-acid -(reaction time 1 min) membrane and **c**, acid fusion solution in methanol (20 ppm) for more than 3days. **b, c** TFC membranes prepared by IP on PAN support.

a b c

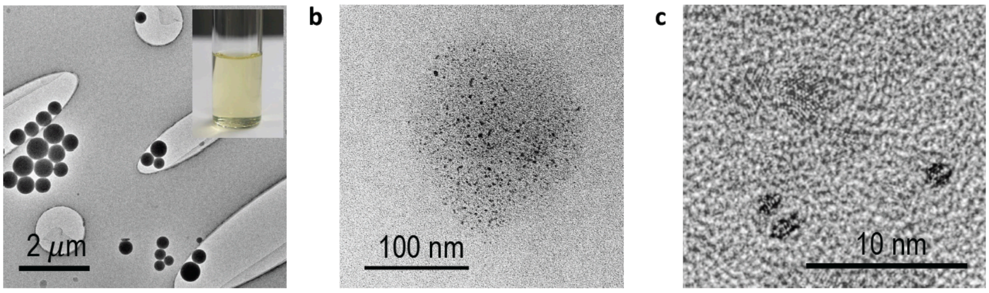

Supplementary Figure 35. HR-TEM micrographs of 0.5wt/v % Pd@RCC3: **a,** densely aggregated RCC3 cages (inset: 0.5 wt/v % solution of Pd@RCC3 in TFE-H_2O mixture used for HR-TEM analysis); **b, c,** non-aggregated Pd@RCC3 and visible lattice fringes of Pd nanoclusters inside the RCC3 cages.

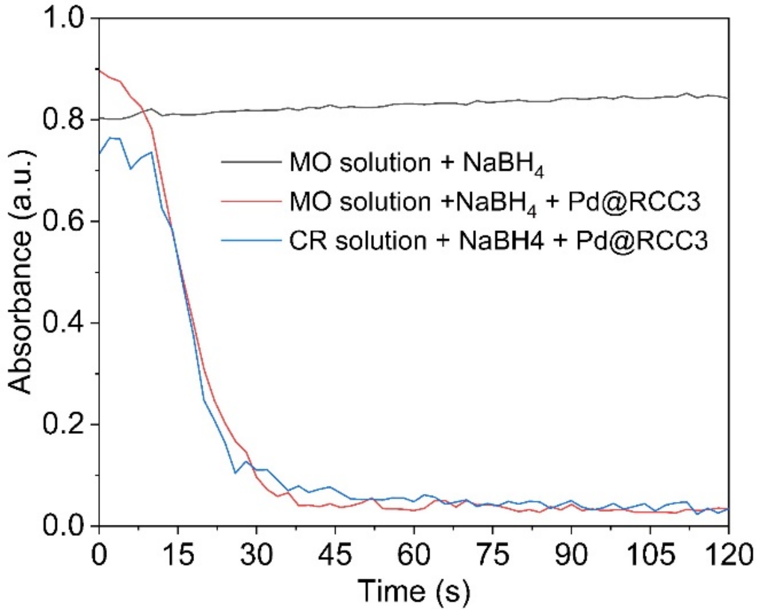

Supplementary Figure 36. Catalytic reduction of organic dyes by using as-synthesized Pd@RCC3 with a reverse double-solvents approach (RDSA).

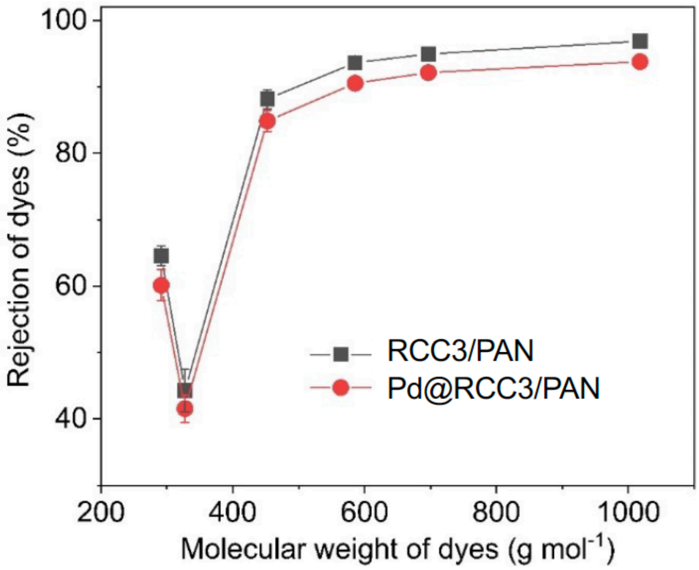

Supplementary Figure 37. Rejection of dyes by RCC3/PAN and Pd@RCC3/PAN membranes. TFC membranes prepared by IP on PAN support.

Supplementary Table 1. Element composition of PAN, cage powders, freestanding polycage nanofilms, and polycage TFC membranes.

	**C (%)**	**O (%)**	**N (%)**	**F (%)**
**PAN**	83.5	0.8	15.7	-
**Powder**				
Tren-cage	93.6	-	6.4	-
RCC3	84.7	8.9	6.4	.
**Free-standing nanofilms**				
Tren-acid	89.3	8.5	2.2	-
Tren-TFE	90.5	7.4	1.4	0.7
RCC3-acid	80.2	15.8	4.0	-
RCC3-TFE	83.1	9.6	6.6	0.7
**TFC membranes**				
Tren-acid	83.6	11.2	5.2	-
Tren-TFE	81.0	9.7	7.8	1.5
RCC3-acid	79.8	8.5	11.7	-
RCC3-TFE	80.1	13.0	6.8	0.1

Supplementary Table 2. Permeance and rejection of selected commercial and lab-scale state-of-the-art membranes.

	Membrane	Name	Permeance (L m^{-2} h^{-1} bar^{-1})				Rejection	Ref.
			MeOH	acetone	THF	toluene		
Commercial membranes	Polyimide	DuraMem® 150	0.48	—	0.10	—	97% (CV/MeOH)	[9,10,7]
	Polyimide	DuraMem® 500	—	—	—	—	95% (AF/ACN)	[11]
	Polyimide	PuraMem® 420	—	—	—	—	98% (AF/ACN)	
	Polyimide	Starmem® 122	—	—	—	0.6	87% (PS/toluene)	[12]
	Polyimide	Starmem® 240	—	—	—	0.7	90% (PS/toluene)	
	Polyimide	Desal-DL	—	—	—	2.6	—	
	Polyimide	Desal-DK	—	—	—	2.6	—	
The state of-the-art membranes	Polyarylate thin film (XP84 support)	PAR-BHPF	8.0	8.4	4.0	0.3	97% (CV/MeOH)	[13]
		PAR-TTSBI	6.0	7.0	4.0	2.5	99% (CV/MeOH)	
		PAR-DHAQ	0.6	0.2	0.04	0.01	98% (CV/MeOH)	
		PAR-RES	0.6	0.4	0.04	0.04	100% (CV/MeOH)	
	Polyamide thin film composite membranes (XP84 support)	MPD-4%-1in-ACT	13.25	12.74	0.18		97.7% (MO/MeOH)	[7]
		MPD-3%-1min	7.71				98.9% (MO/MeOH)	
		MPD-3%-1min-ACT	19.11	32.9			95.5% (MO/MeOH)	
		MPD-0.1%-10min-ACT	9.55	11.3	1.3	0.1	96.8% (MO/MeOH)	
	Polyamide thin film composite	MPD-4%-1in-ACT	34.12	31.16	6.02	1.31	98.8% (MO/MeOH)	
		MPD-3%-1min	13.73	19.34	2.59	0.08	98.9%(MO/MeOH)	

	membranes (alumina support)	MPD-3%-1min-ACT	52.22	49.68	18.11	3.45	98.9%(MO/MeOH)	
		MPD-0.1%-10min-ACT	12.21				99.7%(MO/MeOH)	
	β-CD thin films on PAN support	β-CD-0.1	0.2				92% (MO/MeOH)	14
		β-CD-2.0	9.6				91% (MO/MeOH)	
	CD/TMC membranes on PI support	α-CD/TMC					98.8% (MO/EtOH)	15
		b-CD/TMC	4.9				95.7% (MO/EtOH)	
		r-CD/TMC					94.6% (MO/EtOH)	
	PolyamideCD/PAN membranes	Polyamide-CD (0.64)	17.6	24.26	17	19.8	88% (MO/MeOH)	16
		Polyamide-CD (1.28)	2.45				92% (MO/MeOH)	
	MPCM/PAN thin-film composite membranes	MPCM/PAN(2%, 10s)	22				96% (OG/MeOH)	17
		MPCM/PAN(2%, 10min)	9.3				98% (OG/MeOH)	
		MPCM/PAN(1%, 10s)	39	77.7	51.5	35.5	91% (OG/MeOH)	
		MPCM/PAN(1%, 10min)	21.3				93% (OG/MeOH)	
	Polycage/PAN thin-film composite membranes	Tren-TFE-0.025	0.63				96% (MO/MeOH)	This work
		Tren-TFE-0.07	20.7	39	17.7	15.3	53.9% (MO/MeOH)<br>98.5% (AF/MeOH)	
		Tren-TFE-0.15	66.9				48% (MO/MeOH)<br>94% (AF/MeOH)	
		Tren-acid-10s	9.45				99.4% (AF/MeOH)<br>62% (OGS/MeOH)	

References

1. Lu T, Chen F. Multiwfn: A multifunctional wavefunction analyzer. *Journal of computational chemistry* **33**, 580-592 (2012).

2. Neese F, Wennmohs F, Becker U, Riplinger C. The ORCA quantum chemistry program package. *The Journal of chemical physics* **152**, 224108 (2020).

3. Abbott LJ, Hart KE, Colina CM. Polymatic: a generalized simulated polymerization algorithm for amorphous polymers. *Theoretical Chemistry Accounts* **132**, 1-19 (2013).

4. Plimpton S. Fast parallel algorithms for short-range molecular dynamics. *Journal of computational physics* **117**, 1-19 (1995).

5. Willems TF, Rycroft CH, Kazi M, Meza JC, Haranczyk M. Algorithms and tools for high-throughput geometry-based analysis of crystalline porous materials. *Microporous and Mesoporous Materials* **149**, 134-141 (2012).

6. Van der Bruggen B, Schaep J, Wilms D, Vandecasteele C. Influence of molecular size, polarity and charge on the retention of organic molecules by nanofiltration. *Journal of Membrane Science* **156**, 29-41 (1999).

7. Karan S, Jiang Z, Livingston AG. Sub–10 nm polyamide nanofilms with ultrafast solvent transport for molecular separation. *Science* **348**, 1347-1351 (2015).

8. Buekenhoudt A, Bisignano F, De Luca G, Vandezande P, Wouters M, Verhulst K. Unravelling the solvent flux behaviour of ceramic nanofiltration and ultrafiltration membranes. *Journal of membrane science* **439**, 36-47 (2013).

9. Marchetti P, Jimenez Solomon MF, Szekely G, Livingston AG. Molecular separation with organic solvent nanofiltration: a critical review. *Chemical Reviews* **114**, 10735-10806 (2014).

10. Solomon MFJ, Bhole Y, Livingston AG. High flux membranes for organic solvent nanofiltration (OSN)—Interfacial polymerization with solvent activation. *Journal of membrane science* **423**, 371-382 (2012).

11. Gorgojo P, Karan S, Wong HC, Jimenez‐Solomon MF, Cabral JT, Livingston AG. Ultrathin polymer films with intrinsic microporosity: anomalous solvent permeation and high flux membranes. *Advanced Functional Materials* **24**, 4729-4737 (2014).

12. Othman R, Mohammad AW, Ismail M, Salimon J. Application of polymeric solvent resistant nanofiltration membranes for biodiesel production. *Journal of Membrane Science* **348**, 287-297 (2010).

13. Jimenez-Solomon MF, Song Q, Jelfs KE, Munoz-Ibanez M, Livingston AG. Polymer nanofilms with enhanced microporosity by interfacial polymerization. *Nature Materials* **15**, 760-767 (2016).

14. Villalobos LF, Huang T, Peinemann KV. Cyclodextrin films with fast solvent transport and shape‐selective permeability. *Advanced Materials* **29**, 1606641 (2017).

15. Liu J, Hua D, Zhang Y, Japip S, Chung TS. Precise molecular sieving architectures with Janus pathways for both polar and nonpolar molecules. *Advanced Materials* **30**, 1705933 (2018).

16. Huang T, Puspasari T, Nunes SP, Peinemann KV. Ultrathin 2D‐layered cyclodextrin membranes for high‐performance organic solvent nanofiltration. *Advanced Functional Materials* **30**, 1906797 (2020).

17. Huang T*, et al.* Molecularly-porous ultrathin membranes for highly selective organic solvent nanofiltration. *Nature Communications* **11**, 1-10 (2020).
