# Supplementary material for: Polycage membranes for precise molecular separation and catalysis
Source: Nat Commun. 2023 May 30;14:3112. doi: 10.1038/s41467-023-38728-7 (PMC10229579; doi:10.1038/s41467-023-38728-7)
Supplement: Supplementary file 3 — Description of Additional Supplementary Files [file 41467_2023_38728_MOESM3_ESM.pdf]

Description of Additional Supplementary Files

File Name: Supplementary Movie 1
Description: Molecular visualization of tren cage

File Name: Supplementary Movie 2
Description: Molecular visualization of RCC3 cage

File Name: Supplementary Movie 3
Description: Real-time monitor of the color change of CR dye solution treated with NaBH4 and poly-Pd@RCC3 nanofilms
